# The Complete Mitochondrial Genome of *Squalus cubensis* and Comparative Mitogenomics and Phylomitogenomics of the Family Squalidae

**DOI:** 10.1002/ece3.71412

**Published:** 2025-05-06

**Authors:** Katie Skufca, J. Antonio Baeza

**Affiliations:** ^1^ Department of Biological Sciences Clemson University Clemson South Carolina USA; ^2^ Smithsonian Marine Station at Fort Pierce Smithsonian Institution Fort Pierce Florida USA; ^3^ Departamento de Biología Marina Universidad Catolica del Norte Coquimbo Chile

**Keywords:** genomic resources, mitogenome, phylogenomics, Squalus

## Abstract

The Cuban dogfish, *Squalus cubensis* , is a benthic shark with a discontinuous distribution in the Western Atlantic Ocean, from North Carolina, USA, to San Matias Gulf, Argentina, and is caught in both commercial and recreational fisheries across its range. *Squalus cubensis* is listed as “Least Concern” by the International Union for the Conservation of Nature (IUCN) Red List of Threatened Species. This study generated the first genomic resource for *Squalus cubensis* ; we assembled and characterized its mitochondrial genome in detail. The AT‐rich mitochondrial genome of *Squalus cubensis* is 16,753 base pairs long and contains 22 transfer RNA genes (tRNAs), 2 ribosomal RNA genes (rRNAs), 13 protein‐coding genes (PCGs), and one noncoding control region. Preferential usage of the ATT (Ile), TTA (Leu), and CTA (Leu) codons by PCGs contributes to the AT richness of the mitochondrial genome. Selective pressure analysis indicates that all PCGs encoded in the mitochondrial genome are undergoing purifying selection. Similar to other species within the family Squalidae, the Serine 1 tRNA gene exhibited a truncated D‐arm, as compared to the typical cloverleaf structure of the other 21 tRNA genes. The 1049‐base‐pair‐long control region contains many AT‐rich microsatellite repeats but lacks any tandem repeats. The secondary structure of the control region contains many hairpin loop structures. The completely assembled and analyzed mitochondrial genome of *Squalus cubensis* will assist biomonitoring programs based on environmental DNA (eDNA) and aid in the accurate identification of specimens belonging to the family Squalidae.

## Graphical abstract

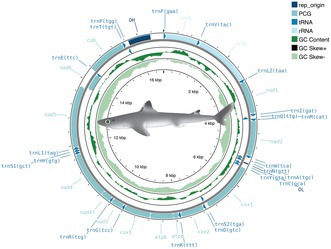

## 1 Introduction

The Cuban dogfish, *Squalus cubensis* (Howell Rivero, 1936), is a benthic shark with a discontinuous geographic range that extends from the Atlantic coast of North Carolina in the United States to Venezuela, and along the eastern coast of South America extending from Rio de Janeiro in Brazil to the San Matias Gulf in Argentina (Cotton et al. 2020). *Squalus cubensis* is active across a wide range of depths and temperatures, typically being found in warmer, shallower waters at night and cooler, deeper waters during the day. Depth measurements for this species based on pop‐up satellite archival tags (PSATs) and catch depth range from 303.9 to 903.8 m and 462 to 730.6 m, respectively (Brooks et al. 2015; Shipley, Howey, et al. 2017).

*Squalus cubensis* is a “K‐selected” species that exhibits biennial aplacental viviparous reproduction and has a fecundity of 1–5 pups per litter (Jones et al. 2013; Tagliafico et al. 2019). There is considerable variation in the regional growth of *S. cubensis* (Brooks et al. 2015), with one stock in the Gulf of Mexico and another in the Bahamas (Pfleger 2016). One estimate reports size at maturity for males in the Bahamas to be 54.5 cm total length (TL) (Brooks et al. 2015), whereas another estimate in the Northern Gulf of Mexico reports maturity of males to be 37.9 cm TL, and maturity of females to be 46.6 cm TL (Jones et al. 2013).

The diet of *Squalus cubensis* primarily consists of teleost fish and squid (Churchill et al. 2015), with its diet changing from lower to higher trophic level prey throughout its lifetime (Shipley, Polunin, et al. 2017). Limited predation data of *S. cubensis* report it to be consumed by other sharks, including the silky shark *Carcharhinus falciformis* and giant isopod *Bathynomus giganteus* (Talwar et al. 2017).

*Squalus cubensis* are caught incidentally via electric reel and fish trap in shark fisheries in Puerto Rico (Espinoza et al. 2024), opportunistically caught via vertical and hand lines in pelagic longline fisheries in deep water off Northwestern Cuba (Ruiz‐Abierno et al. 2021), and intentionally and indiscriminately caught for fins, with the carcass byproduct being sold for consumption in Brazilian fish markets (Almerón‐Souza et al. 2018). In addition to targeted fisheries for *S. cubensis* , *Squalus* species are exploited for their livers, which contain compounds that are used in commercial products. One of these compounds is Squalene, which is used in cosmetics and dietary supplements (Cruz‐Nuñez et al. 2009). Another one of these compounds is Squalamine, which has been found to contain antiangiogenic and antimicrobial properties and has previously been implemented in trials testing potential treatments for macular degeneration, retinopathy, Parkinson disease, Alzheimer disease, and various types of cancer (Bhargava et al. 2001; Emerson and Lauer 2008; Limbocker et al. 2022).

There is limited data on the exact number of *Squalus cubensis* individuals caught by fisheries, but the combined effect of overfishing for deepwater sharks for use in the shark liver oil and shark meat trade is cause enough for concern (Finucci et al. 2024). Postcatch at‐vessel mortality rates are higher for smaller‐bodied benthic sharks, with *S. cubensis* having a postcatch mortality rate of 9.09%, and a post‐release mortality (PRM) rate of 49.7% (Braccini and Waltrick 2019; Brooks et al. 2015; Talwar et al. 2017). *Squalus cubensis* is listed as Least Concern by the IUCN Red List, with an increasing population trend (Cotton et al. 2020). Despite current population trends for the species implying that current use is sustainable, the K‐selected life history traits of this species make it more vulnerable to exploitation, and prior studies warn against expansion of shark fisheries within this species native range (Bonfil 1997; Jones et al. 2013).

Many elasmobranch species, including *Squalus cubensis* , are sold for meat under the general name “cação” in Southern Brazilian fish markets and are typically sold with identifying characteristics removed, making it impossible to identify carcasses without DNA testing, which opens up the possibilities for illegal trading of endangered or vulnerable shark species (Almerón‐Souza et al. 2018). There is further concern in identifying specimens across international trade due to a *Squalus cubensis* specimen found in a Mediterranean fish market, which is far from the Western Atlantic region where this species is found in (Giovos et al. 2021). The issue of identifying species is further complicated by the sale of specimens that have been exposed to human‐caused toxins, such as *Squalus cubensis* being exposed to polycyclic aromatic hydrocarbons during the Deepwater Horizon oil spill (Leary 2015).

Despite the importance of *Squalus cubensis* in fisheries, human disease treatments, and the concerns regarding species identification both in and outside of its range, there is no genomic resource for this species and only limited genetic data. Other genomic resources of varying complexity exist for other species within the family Squalidae, including *Squalus brevirostris* (Zhang et al. 2019), *Squalus formosus* (Chen et al. 2016), *Squalus blainville* (Kousteni et al. 2021), *Squalus montalbani* (Kemper and Naylor 2016), and *Cirrhigaleus australis* (Yang et al. 2016). Generating genomic resources is important for understanding populations of species and identifying unknown species when physical characteristics are removed.

The primary goal of this study was to assemble and annotate the entire mitochondrial genome of *Squalus cubensis* in detail using various genomic tools. The detailed characterization methods provided by Baeza (2022) were followed. In addition to characterizing the mitochondrial genome, we also investigated the phylogenetic position of *S. cubensis* within the family Squalidae based on the phylogenetic signal provided by translated protein‐coding genes (PCGs).

## 2 Materials and Methods

We sequenced the mitochondrial genome using DNA extracted from the *Squalus cubensis* specimen USNM400722, belonging to the fish collection of the National Museum of Natural History, Smithsonian Institution, Washington DC, USA. The specimen was collected in the Caribbean Sea, near Panama (8.97005, −77.4572) on January 6, 2011.

Following Cady et al. (2021), gDNA was extracted from muscle tissue using the extraction robot AutoGenPrep 965 automated DNA (AutoGen, Holliston, MA, USA). Next, as in Cady et al. (2021), Illumina paired‐end shotgun libraries were prepared using the standard NEB Ultra II DNA library prep kit protocol (New England Biolabs, Ipswich, MA, USA). Then, sequencing of the prepared library was conducted on an Illumina MiSeq (Illumina, San Diego, CA, USA) using 2 × 150 cycles. A total of 8,371,252 pairs of reads were used for assembling the mitochondrial genome of *Squalus cubensis* with the software GetOrganelle (Jin et al. 2020) and using the mitochondrial genome of *Squalus blainville* as a seed (GenBank Accession Number: NC_059940).

The assembled mitochondrial genome was first annotated using the pipeline MITOS2 as implemented in the platforms GalaxyEuro (Arab et al. 2017; Donath et al. 2019) and Proksee (https://proksee.ca—Grant et al. 2023). A circular depiction of the mitochondrial genome was generated using the web server Proksee (https://proksee.ca—Grant et al. 2023). The nucleotide usage of the entire mitochondrial genome and specific genes was calculated using the software MEGA 11 (Tamura et al. 2021).

The Codon Usage tool within the Sequence Manipulation Suite web server was used to determine the codon usage for all 13 PCGs within the mitochondrial genome (https://www.bioinformatics.org/sms2/codon_usage.html—Stothard 2000). The EZcodon tool within EZmito was used to determine the relative synonymous codon usage within the 13 PCGs (http://ezmito.unisi.it/ezcodon—Cucini et al. 2021; Lee 2018).

In order to conduct selection pressure analysis on the PCGs within the mitochondrial genome of *Squalus cubensis* , we first needed to align PCG sequences from *S. cubensis* and another closely related species to determine the Ka (nonsynonymous substitution rate)‐to‐Ks (synonymous substitution rate) ratio. To conduct this analysis, the mitochondrial genome of *Squalus montalbani* [GenBank: KT459334] was chosen, which was previously annotated using the MITOS2 tool within the GalaxyEuro web server in order to determine the sequences of the PCGs (Arab et al. 2017; Donath et al. 2019). We then aligned the PCG sequences from each species by MUSCLE (codons) within the Mega 11 software (Tamura et al. 2021). The aligned sequences were then uploaded into the KaKs_calculator 2.0 program using the gMYN method to account for variation in substitution rates (Wang et al. 2009; Wang et al. 2010). PCGs are presumed to be under neutral selection when Ka/Ks = 1, positive/diversifying selection when Ka/Ks > 1, and negative/purifying selection when Ka/Ks < 1 (Li et al. 2009). Associated *p* values were expected to be < 0.05 for the data to be considered statistically significant.

The secondary structure of each tRNA gene in the mitochondrial genome was predicted using the MITFI program included in the MITOS2 program (Arab et al. 2017; Donath et al. 2019; Jühling et al. 2012). MITFI secondary structure predictions were then visualized using the Forna RNA Secondary Structure visualization tool available in the web server ViennaRNA Web Services (http://rna.tbi.univie.ac.at/forna/—Kerpedjiev et al. 2015).

In order to characterize in detail the control region of the studied mitochondrial genome, we used the BioPHP Microsatellite Repeat Finder tool with default parameters to determine the presence/absence, number, and identity of microsatellites (http://insilico.ehu.es/mini_tools/microsatellites/—Bikandi et al. 2004). Additionally, the web server Tandem Repeats Finder (https://tandem.bu.edu/trf/trf.html—Benson 1999) was used to search for possible tandem repeats within the control region. Finally, the secondary structure of the CR was predicted using the default parameters on the RNAfold web server (http://rna.tbi.univie.ac.at/cgi‐bin/RNAWebSuite/RNAfold.cgi—Gruber et al. 2008; Lorenz et al. 2011).

### 2.1 The Phylogenetic Position of *Squalus cubensis*

We examined the phylogenetic position of *Squalus cubensis* within the family Squalidae and the order Squaliformes. The newly assembled mitochondrial genome of *Squalus cubensis* , along with 6 other mitochondrial genomes belonging to the family Squalidae (*Squalus* [*n* = 5 species], *Cirrhigaleus* [*n* = 1]), 11 mitochondrial genomes from other representatives of the order Squaliformes (Oxynotidae [*n* = 1 species], Somniosidae [*n* = 2], Dalatiidae [*n* = 3], Etmopteridae [*n* = 3], and Centrophoridae [*n* = 2]), and 11 mitochondrial genomes from the superorder Squalomorphii (Echinorhiniformes [*n* = 1], Hexanchiformes [*n* = 3], Pristiophoriformes [*n* = 1], and Squatiniformes [*n* = 7]) were used for maximum‐likelihood phylogenetic inference. All mitochondrial genomes used are available in GenBank. First, we retrieved all 13 PCG nucleotide sequences from each mitochondrial genome and translated the sequences into amino acids using the program MEGA 11 (Tamura et al. 2021). Then, each of the amino acid sequences was aligned using the software Clustal Omega (Sievers and Higgins 2014) as implemented in MEGA 11. Subsequently, poorly aligned regions in each amino acid alignment were extracted with trimAl (Capella‐Gutiérrez et al. 2009) and the best‐fitting model of sequence evolution for each PCG was chosen using the program Protest (Darriba et al. 2011). Finally, the partitioned and concatenated PCG amino acid–aligned dataset was used to perform an ML analysis using the web portal IQ‐TREE version 1.6.10 (Nguyen et al. 2015). The robustness of the ML tree topology was evaluated by 1,000 bootstrap repetitions of the dataset.

We additionally conducted a Bayesian Inference (BI) phylogenetic analysis in the program MrBayes 3.2.1 (Ronquist et al. 2012). The analysis extended 6,000,000 generations, and our exploration of log‐likelihood scores over generations displayed a stable equilibrium prior to the 100,000th generation. Thus, a burn‐in phase of 1,000 samples was conducted, and every 100th tree was sampled from the Metropolis‐coupled Markov Chain Monte Carlo generating a sum of 60,000 trees. A consensus tree, abiding by the 50% majority rule, was obtained from the last 59,000 sampled trees (Ronquist et al. 2012).

## 3 Results and Discussion

**FIGURE 1:**
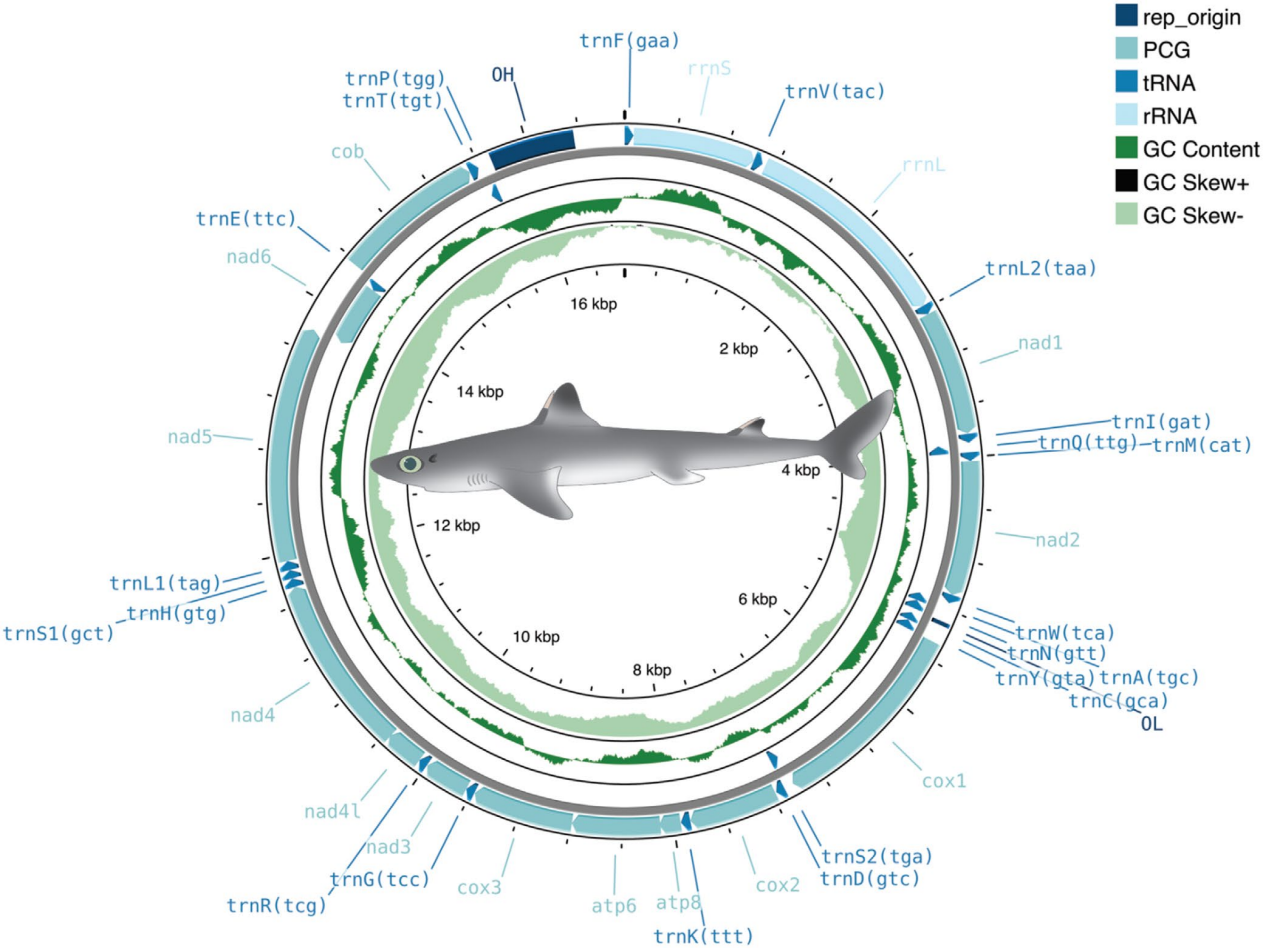
Circular depiction of the mitochondrial genome of *Squalus cubensis* . Illustration credit: Katie Skufca.

The complete mitochondrial genome of *Squalus cubensis* [GenBank: OP056876] is 16,753 base pairs (bp) long and contains 22 transfer RNA genes (tRNAs), 2 ribosomal RNA genes (rRNAs), 13 protein coding genes (PCGs), and one noncoding control region. Of the 37 genes found in the mitochondrial genome of *Squalus cubensis* , 28 genes are encoded on the heavy strand, and eight tRNAs and one PCG (*nad6*) are encoded on the light strand (Figure 1). Gene composition and order are identical to that of all other reported species within the family Squalidae (Chen et al. 2016; Kemper and Naylor 2016; Kousteni et al. 2021; Yang et al. 2016; Zhang et al. 2019). Additionally, the length of the studied mitochondrial genome is similar to that reported before for other cofamilial species. Mitochondrial genome length in the family Squalidae ranges from 16,544 base pairs in *Cirrhigaleus australis* (Yang et al. 2016) to 16,753 base pairs in *Squalus cubensis* . Arrangement and annotation of the mitochondrial genome of *Squalus cubensis* can be found in Table 1.

**TABLE 1:** Mitochondrial genome of *Squalus cubensis* . Arrangement and annotation.

Name	Type	Start	Stop	Strand	Length (bp)	Start	Stop	Anticodon	Continuity
trnF	tRNA	1	68	+	68			GAA	1
rrnS	rRNA	70	1022	+	953				−3
trnV	tRNA	1020	1091	+	72			TAC	25
rrnL	rRNA	1117	2769	+	1653				−1
trnL2	tRNA	2769	2843	+	75			TAA	0
nad1	PCG	2844	3818	+	975	ATG	TAA		3
trnI	tRNA	3822	3891	+	70			GAT	1
trnQ	tRNA	3893	3964	−	72			TTG	0
trnM	tRNA	3965	4033	+	69			CAT	0
nad2	PCG	4034	5080	+	1047	ATG	TAA		−1
trnW	tRNA	5080	5148	+	69			TCA	1
trnA	tRNA	5150	5218	−	69			TGC	0
trnN	tRNA	5219	5292	−	74			GTT	6
OL	RepOrigin	5299	5329	+	31				1
trnC	tRNA	5331	5397	−	67			GCA	1
trnY	tRNA	5399	5468	−	70			GTA	1
cox1	PCG	5470	7026	+	1557	GTG	TAA		0
trnS2	tRNA	7027	7097	−	71			TGA	2
trnD	tRNA	7100	7169	+	70			GTC	8
cox2	PCG	7178	7868	+	691	ATG	T		0
trnK	tRNA	7869	7942	+	74			TTT	1
atp8	PCG	7944	8111	+	168	ATG	TAA		−10
atp6	PCG	8102	8785	+	684	ATG	TAA		−1
cox3	PCG	8785	9570	+	786	ATG	TAA		2
trnG	tRNA	9573	9642	+	70			TCC	0
nad3	PCG	9643	9993	+	351	ATG	TAA		3
trnR	tRNA	9997	10066	+	70			TCG	0
nad4l	PCG	10067	10363	+	297	ATG	TAA		−7
nad4	PCG	10357	11737	+	1381	ATG	T		0
trnH	tRNA	11738	11806	+	69			GTG	0
trnS1	tRNA	11807	11873	+	67			GCT	0
trnL1	tRNA	11874	11945	+	72			TAG	0
nad5	PCG	11946	13778	+	1833	ATG	TAA		−4
nad6	PCG	13775	14296	−	522	ATG	TAG		0
trnE	tRNA	14297	14366	−	70			TTC	4
cob	PCG	14371	15516	+	1146	ATG	TAA		1
trnT	tRNA	15518	15590	+	73			TGT	2
trnP	tRNA	15593	15661	−	69			TGG	43
OH	RepOrigin	15705	16,362	+	658				

The nucleotide usage of the complete mitochondrial genome of *Squalus cubensis* is 30.0% T, 30.9% A, 24.8% C, and 14.3% G, with an A + T content of 60.9%. A + T usage in the mitochondrial genome of the family Squalidae ranges from 60.89% in *Squalus blainville* (Kousteni et al. 2021) to 61.4% in *S. formosus* (Chen et al. 2016). Nucleotide usage for *Squalus cubensis* is similar to that of other species within the family Squalidae. *Squalus brevirostris* was reported to exhibit an A + T content reversed from what has been observed in congeneric species: A = 30.38% and T = 30.73% (Zhang et al. 2019). We retrieved the mitochondrial genome of *Squalus brevirostris* [KY111436] from GenBank, recalculated the nucleotide usage of this species in the program MEGA11, and found that the nucleotide usage was within the expected range of values for other members of the Squalidae family, with an A usage of 30.70% and a T usage of 30.40%. Nucleotide usage for the Squalidae family can be found in Table S1, along with a heatmap comparison of nucleotide usage found in Figure S1.

The 13 PCGs in the mitochondrial genome of *Squalus cubensis* range in length from 168 bp (*atp8*) to 1,833 bp (*nad5*), similar to that reported for other closely and distantly related sharks (Kousteni et al. 2021; Yang et al. 2016; Zhang et al. 2019). Of the 13 PCGs in the *Squalus cubensis* mitogenome, all PCGs except *cox1*, which uses GTG as its start codon, exhibited ATG as the start codon. Most of the PCGs have TAA as the stop codon, except for Cox2, Nad4, and Nad6. Cox2 and Nad4 have an incomplete stop codon of T, and Nad6 has the TAG stop codon. Start and stop codon usage in the studied species is identical to that of *Squalus formosus* (Chen et al. 2016). Start and stop codon usage differs slightly between the studied species and *Squalus blainville* , which is reported as having CTA and T‐ as the respective start and stop codons for Nad6 (Kousteni et al. 2021). *Cirrhigaleus australis* differs from *Squalus cubensis* in that NAD5 uses the TAG stop codon in addition to NAD6 (Yang et al. 2016).

The most commonly used codons within the mitochondrial PCGs of *S. cubensis* were ATT (Ile, *n* = 215, 5.65% total), TTA (Leu, *n* = 202, 5.299% total), and CTA (Leu, *n* = 175, 4.591% total). The least commonly used codons were TAG (Stop, *n* = 1, 0.026% total) and GCG (Ala, *n* = 4, 0.105% total), CGG (Arg, *n* = 4, 0.105% total), and TCG (Ser, *n* = 4, 0.105% total). PCGs make up 11,438 base pairs of the 16,753 base pair mitochondrial genome. The only other species within the Squalidae family with reported codon usage analysis is *Squalus blainville* , which has similar usage to *Squalus cubensis* in that ATT (Ile, *n* = 168) and TTA (Leu, *n* = 156) are the two most used codons but differs in that its third most used codon is TTT (Phe, *n* = 148) (Kousteni et al. 2021). The least used codons in *Squalus blainville* are similar to *Squalus cubensis* , with GCG (Ala, *n* = 6) and CGG (Arg, *n* = 13) being two of its least used codons, but differs from *Squalus cubensis* in that CGT (Arg, *n* = 13) is tied for its third least used codon (Kousteni et al. 2021). Frequent usage of AT‐rich codons in *Squalus cubensis* and other *Squalus* species may contribute to the AT‐rich mitochondrial genomes seen in the family Squalidae.

**FIGURE 2:**
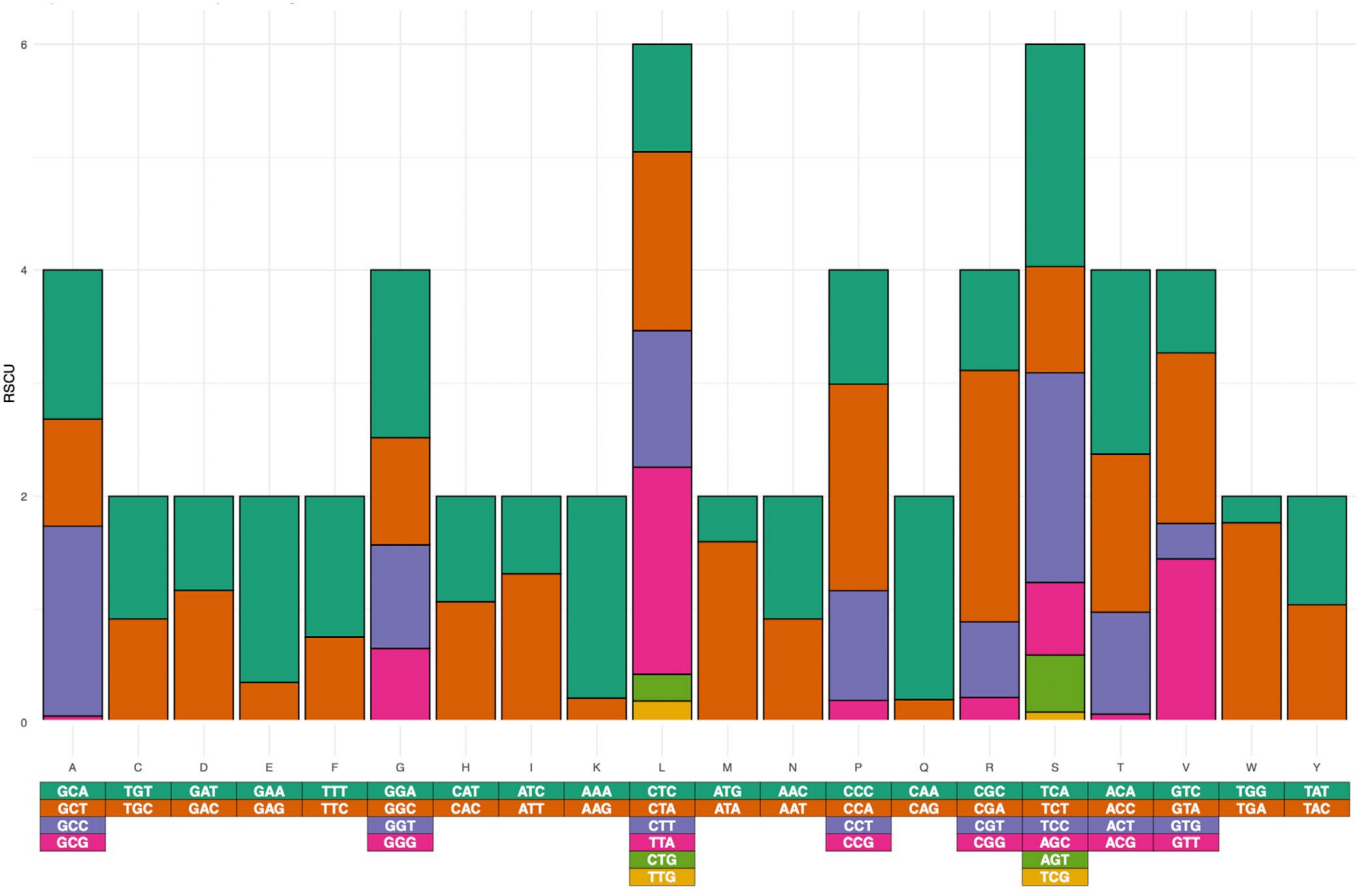
Relative synonymous codon usage within the PCGs of *Squalus cubensis*.

The relative synonymous codon usage (RSCU) analysis for the PCGs within the mitochondrial genome of *Squalus cubensis* found that the highest RSCU values were for CGA (Arginine, 2.222), TCA (Serine, 1.969), and TCC (Serine, 1.855). The lowest RSCU values were for GCG (alanine, 0.056), ACG (threonine, 0.073), and TCG (serine, 0.092) (Figure 2). The highest RSCU in *Squalus blainville* differs significantly from *Squalus cubensis* , having high RSCU values for GUU (valine, 1.70), UUA (leucine, 1.57), GAA (glutamic acid, 1.49), GCC (alanine, 1.49), and CAA (glutamine, 1.49) (Kousteni et al. 2021). The lowest RSCU in *Squalus cubensis* and *Squalus blainville* are similar, with *S. blainville* having low values for GCG (alanine, 0.13), ACG (threonine, 0.26), and CCG (proline, 0.35) (Kousteni et al. 2021). Preferential usage of codons that are AT rich in the second or third position, in comparison to other synonymous codons, is common across other elasmobranch species (Kousteni et al. 2021).

Selective pressure analysis of PCGs within the mitochondrial genome of *Squalus cubensis* revealed that all 13 PCGs had a Ka/Ks value of less than one (all associated *p* values were < 0.05), indicating strong negative/purifying selection (Table 2). All Ka/Ks values fell within the range of 0.00248 (Cox1) to 0.0762 (Nad4l), except for Cox2 and Nad3, which exhibited Ka/Ks values of 1.32 × 10^−14^ and 3.41 × 10^−15^, respectively. A table of the Ka, Ks, Ka/Ks, and *p* values can be found in Table 2. This indicates stronger purifying selection within these two genes, due to the values being closer to zero. This is the first study of selective pressure analysis of mitochondrial PCGs within the family Squalidae. There is limited selective pressure analysis of PCGs in mitochondrial genomes of chondrichthyans, but studies on the lemon shark ( *Negaprion brevirostris* ), Japanese swellshark (*Cephaloscyllium umbratile*), and smoothhound sharks ( *Mustelus canis* and *Mustelus norrisi* ) have indicated that PCGs in the aforementioned species undergo strong purifying selection (Baeza et al. 2024; Kiser et al. 2024; Zhu et al. 2017). The *cox1* gene has some of the lowest Ka/Ks values across all five species, indicating strong purifying selection for the Cox gene families. Atp8 has the highest Ka/Ks value for *Cephaloscyllium umbratile*, *Negaprion brevirostris* , *Mustelus canis* , and *Mustelus norrisi* , with values of 0.5322, 0.18, 0.142, and 0.114, respectively (Baeza et al. 2024; Kiser et al. 2024; Zhu et al. 2017). *Squalus cubensis* differs from these species by having stronger purifying selection in its atp8 gene, with a Ka/Ks value of 0.028. *Squalus cubensis* also differs from the Ka/Ks values found in *Negaprion brevirostris* , which has stronger purifying selection in many of its Nad genes (Zhu et al. 2017), whereas *Squalus cubensis* has more relaxed purifying selection in its Nad genes compared to the Ka/Ks values of its other genes. Previous studies indicate that the PCGs in the mitochondrial genomes of sharks and other vertebrates are typically subjected to strong purifying selection (Baeza et al. 2024).

**TABLE 2:** Selective pressure analysis of the protein‐coding genes within the mitochondrial genome of *Squalus cubensis* . Ka/Ks values were calculated using the ‐gMYN method using the mitochondrial genome of *Squalus montalbani* as a comparison.

Gene	*K*_*a*_	*K*_*s*_	*K*_*a*_/*K*_*s*_	*p*
*atp6*	0.00599737	0.280944	0.0213472	9.63E‐17
*atp8*	0.0148767	0.524464	0.0283655	1.68E‐06
*cob*	0.0131678	0.384137	0.0342789	6.42E‐39
*cox1*	0.000840021	0.33877	0.00247962	1.46E‐60
*cox2*	2.22E‐15	0.168012	1.32E‐14	0
*cox3*	0.00161627	0.248812	0.00649593	3.38E‐22
*nad1*	0.00422498	0.229263	0.0184285	9.31E‐26
*nad2*	0.0113531	0.222922	0.0509286	1.51E‐20
*nad3*	1.11E‐15	0.32592	3.41E‐15	0
*nad4*	0.0078212	0.381713	0.0204898	4.61E‐46
*nad4l*	0.0150897	0.197924	0.0762396	4.48E‐06
*nad5*	0.0158343	0.261058	0.0606541	3.08E‐42
*nad6*	0.0113172	0.207755	0.0544737	1.23E‐09

**FIGURE 3:**
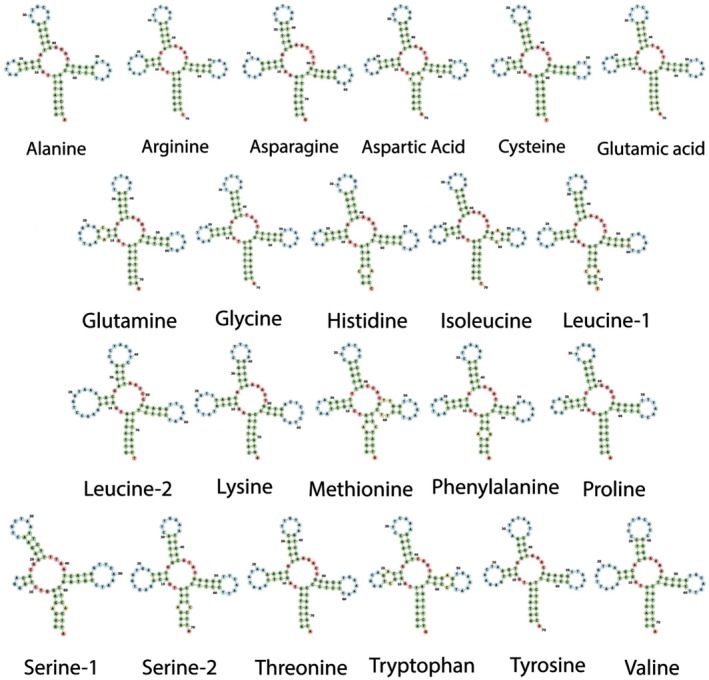
Visualizations of the tRNA genes within the mitochondrial genome of *Squalus cubensis*.

Of the 22 transfer RNAs within the mitochondrial genome of *Squalus cubensis* , the longest tRNA was Leucine 2 (75 bp), and the shortest tRNAs were Cysteine and Serine 1 (67 bp). The shortest and longest tRNA in other species within the family Squalidae are the same (Kousteni et al. 2021; Yang et al. 2016). Each of the tRNAs in the *Squalus cubensis* mitogenome had a typical cloverleaf structure expected of most tRNA genes, except for the Serine 1 tRNA that exhibited a simple loop in place of the dihydrouridine (DHU) arm (Figure 3). This truncation is reported in other species within the genus *Squalus*, including *Squalus blainville* , and has been reported in other chondrichthyans (Kousteni et al. 2021). *Squalus formosus* is reported as having Serine 2 lacking a typical cloverleaf structure, rather than Serine 1 (Chen et al. 2016). Truncation of the D‐arm in one or both serine genes is common across Eumetazoans and Elasmobranchs (Bernt et al. 2013; Boore et al. 2005; Kousteni et al. 2021). Functionality of the truncated serine genes in the Squalidae family and other vertebrates remains to be addressed, but some studies suggest that truncated tRNAs are useful in assisting with translation (Watanabe et al. 2014).

**FIGURE 4:**
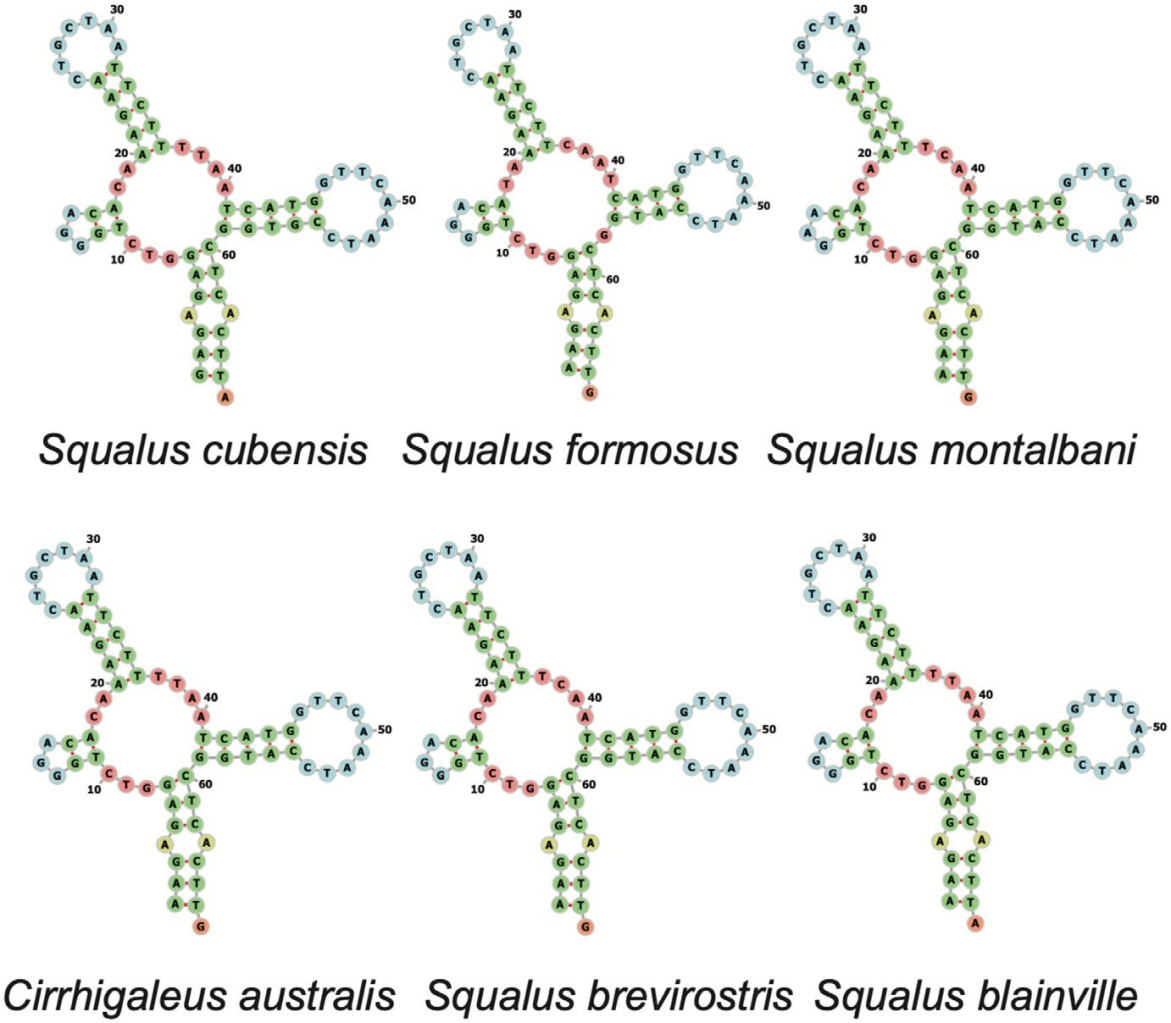
Serine 1 tRNA genes within the Squalidae family.

**FIGURE 5:**
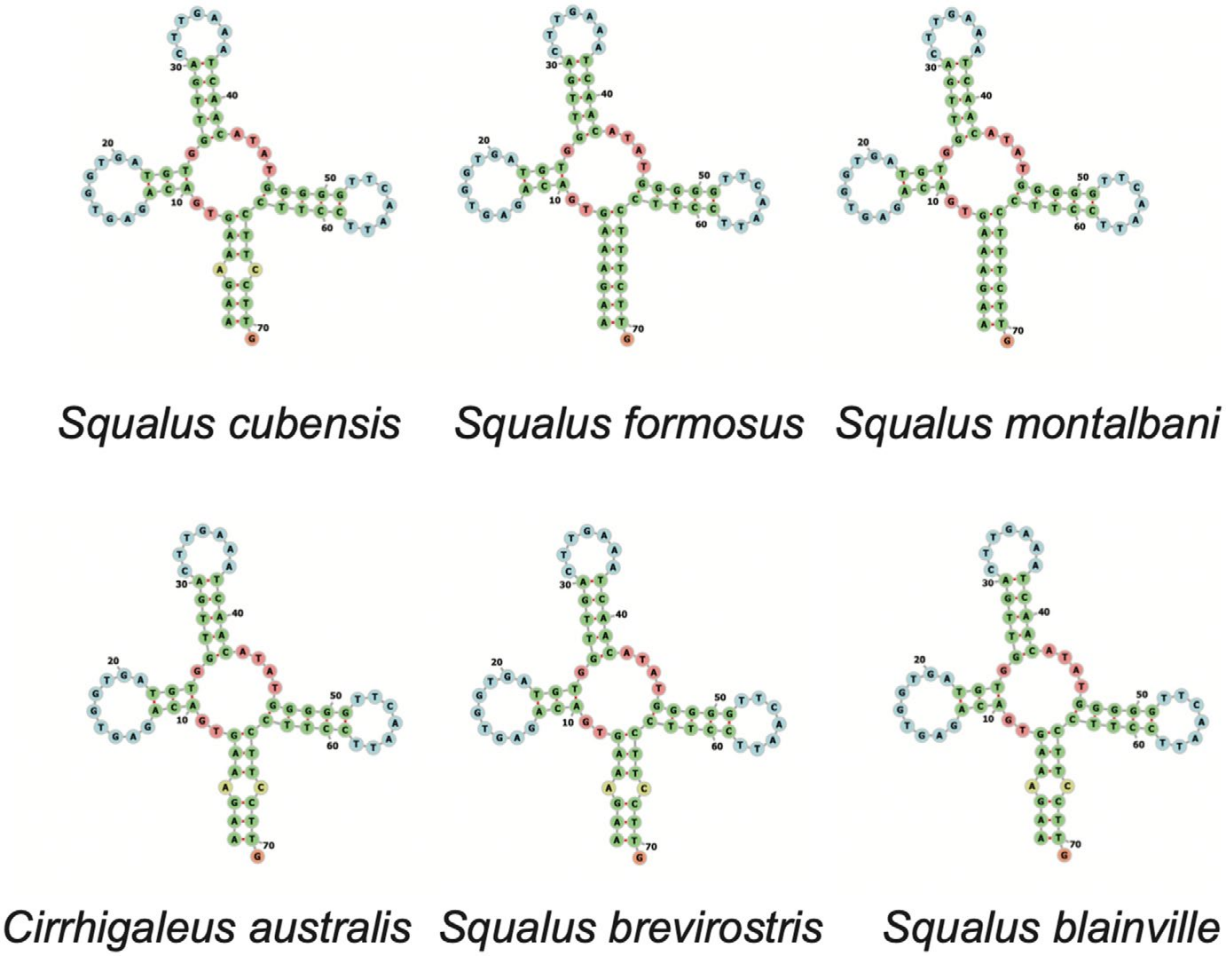
Serine 2 tRNA genes within the Squalidae family.

There is no standard nomenclature for leucine and serine genes (Bernt et al. 2013), which leads to different species within the same genus being reported as having different serine genes as truncated. Due to this discrepancy, we generated visualizations of the tRNA‐S1 and tRNA‐S2 genes across the five other reported species in the Squalidae family in order to determine if different papers were using inconsistent naming systems for naming the tRNA‐S1 and tRNA‐S2 genes. Mitos2 names the first listed serine gene, the one located between Cox1 and tRNA Asp as “Serine 2” (See Table 1), whereas other papers may be calling the second listed serine, the one located between tRNA His and tRNA Leu, as “Serine 2”; which leads to some confusion on which serine gene is truncated. Having a consistent naming system is essential to understanding and comparing genome analysis (Bernt et al. 2013). After acquiring the mitogenomes from GenBank for the additional five published genomes for species within the family Squalidae ( *Squalus formosus* [GenBank: KU951280], *S. montalbani* [GenBank: KT459334], *S. brevirostris* [KY111436], *S. blainville* [MT274575], and *Cirrhigaleus australis* [KJ128289]), we used Mitos2 annotation and Forna tRNA visualization to determine the secondary structure of the Serine 1 and Serine 2 gene for each species. We found that all six reported species within the Squalidae family have a truncated tRNA Serine 1 that lacks a DHU arm, with “Serine 1” being distinguished as the serine located between tRNA His and tRNA Leu. The Serine 2 gene, distinguished as the serine located between Cox1 and tRNA Asp, in all six species, exhibits a typical cloverleaf structure. The 2D structure of both Serine 1 and Serine 2 in all six compared species can be found in Figures 4 and 5.

There are two ribosomal RNA genes within the mitochondrial genome of *Squalus cubensis* , the small (12S) and large (16S) ribosomal RNAs, which consist of 953 and 1,653 base pairs, respectively. 12S rRNA length ranges from 951 bp ( *Squalus formosus* , *S. blainville* ) to 954 bp (*Cirrhigaleus australis*). (Chen et al. 2016; Kousteni et al. 2021; Yang et al. 2016). The 16S rRNA length ranges from 1,653 bp ( *S. cubensis* ) to 1,678 bp (*Cirrhigaleus australis*) (Yang et al. 2016). The nucleotide usage for the rRNA genes in *Squalus cubensis* is 26.5% T, 21.1% C, 34.4% A, and 18.0% G, with an AT skew of 60.9%, which is reflective of the AT skew of 60.9% of the entire mitochondrial genome. There is no reported information on the nucleotide usage within the rRNA genes of other species within the Squalidae family.

The control region of the mitochondrial genome of *Squalus cubensis* is 1,049 base pairs long and is located between the trna‐Pro and trna‐Phe. Within the control region is the 658 base pair heavy strand origin of replication (OH). The AT content of the control region is 66.8%, a higher AT content compared to the entire mitochondrial genome. Within the control region, there were 10 microsatellite repeats, most of which were AT‐rich dinucleotide repeats (Table S2). The high percentage of AT and TT microsatellites is reflective of the AT skew of the control region. There were no tandem repeats found within the control region. The lack of tandem repeats is not unexpected, as it has also been reported in the congeneric species *Squalus blainville* (Kousteni et al. 2021). The secondary structure of the control region in *Squalus cubensis* contains many stem loops across the entire sequence (Figure S2). Control region length varies slightly between members of the Squalidae family, ranging from 885 base pairs in Cirrhigaleus *australis* to 1,096 base pairs in *Squalus blainville* (Kousteni et al. 2021; Yang et al. 2016). The AT content of the control region in other reported species is similar to that of *Squalus cubensis* , with *Squalus formosus* having a reported AT content of 66.9% (Chen et al. 2016). The location of the control region in *Squalus cubensis* is the same in other reported cofamilial species (Chen et al. 2016; Kousteni et al. 2021; Yang et al. 2016; Zhang et al. 2019). The function of the control region within the mitochondrial genome remains to be addressed through further comparative studies.

### 3.1 The Phylogenetic Position of *Squalus cubensis*

**FIGURE 6:**
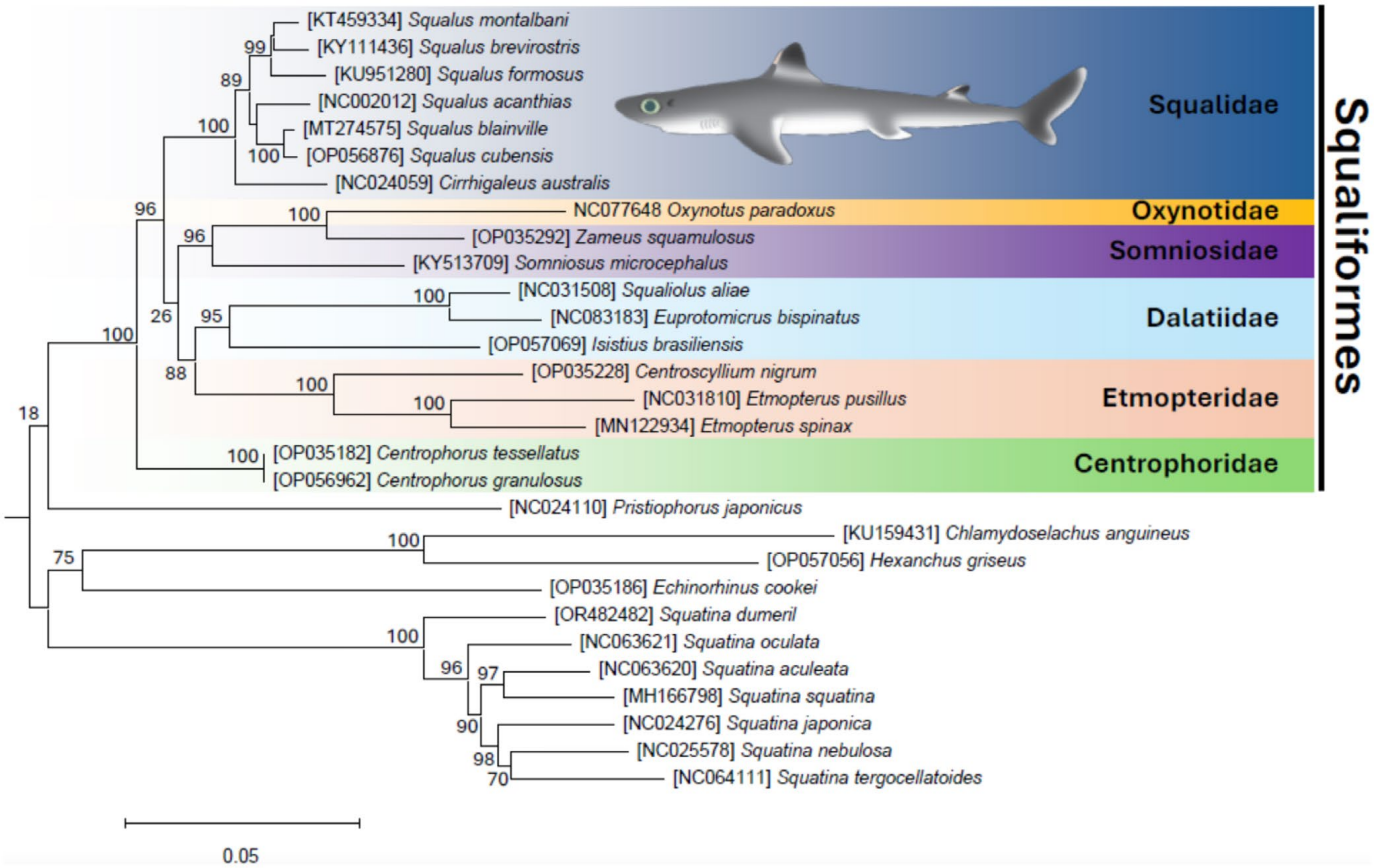
Phylomitogenomic tree created from ML analysis based on a concatenated alignment of amino acids from the 13 PCGs found in the *Squalus cubensis* mitochondrial genome, and other species from the Squaliformes order. Eleven additional mitochondrial genomes from superorder Squalomorphii were used as outgroups, including members from Echinorhiniformes (*n* = 1), Hexanchiformes (*n* = 3), Pristiophoriformes (*n* = 1), and Squatiniformes (*n* = 7). Accompanying numbers are the GenBank accession numbers for the sequences used.

The ML analysis (29 terminals, 3801‐amino‐acid characters, and 814 informative sites) fully supported (bootstrap value [bv] = 100) the order Squaliformes as monophyletic (Figure 6).

The ML analysis also supported the monophyletic status of the families Centrophoridae (represented by two species belonging to the genus *Centrophorus* in our analysis), Dalatiidae (represented by three different genera), and Etmopteridae (represented by three species belonging to two genera). The family Somniosidae was not supported as monophyletic considering that *Oxynotus paradoxus* , belonging to the family Oxynotidae, formed a fully supported clade with *Zameus squamulosus* (family Somniosidae) that, in turn, was well supported (bv = 96) as sister to *Somniosus microcephalus* , in the family Somniosidae. The family Centrophoridae was supported as a clade sister to all other representatives of the order Squaliformes in our analysis, but other family‐level relationships were not recovered by our analysis.

In the ML phylogenetic tree, the family Squalidae was fully supported as monophyletic. Within this latter family, the Southern Mandarin dogfish *Cirrhigaleus australis* was sister to a moderately supported clade (bv = 89) containing all species of *Squalus* used in the analysis. The studied species, *S. cubensis* , was fully supported as a sister to *S. blainville* in our ML analysis.

The genus *Squalus* represents a taxonomically complicated and understudied clade (Veríssimo et al. 2017) with a few previous studies not supporting its monophyletic status. For instance, using limited datasets [compared to the present study], Chen et al. (2016), Kemper and Naylor (2016), Zhang et al. (2019), and Kousteni et al. (2021) found *Cirrhigaleus australis* to cluster deep within a clade comprising species of *Squalus*. On the other hand, in agreement with our results, Ferrari et al. (2021) and Veríssimo et al. (2017) supported the monophyletic status of the genus *Squalus* and recovered *Cirrhigaleus australis* as its sister clade.

In order to continue probing the monophyletic status of the family Squalidae and to improve our understanding of the evolutionary relationships within this family and the genus *Squalus*, we argue in favor of additional studies assembling and characterizing in detail the mitochondrial genome and sequencing nuclear markers for representatives of the order Squaliformes.

## 4 Conclusion

In this study, we assembled and characterized in detail the complete mitochondrial genome of *Squalus cubensis* . We found minimal differences between *Squalus cubensis* and other species within the family Squalidae, including the most and least frequently used codons differing slightly from *Squalus blainville* , in addition to small variations in AT content and control region length. In addition to the differences we found, there were many similarities between *Squalus cubensis* and other cofamilial species, including all reported species having a truncated D‐arm on the Serine 1 tRNA gene, lacking tandem repeats within the control region, and shortest and longest PCGs and tRNAs. Analysis of selective pressure in *Squalus cubensis* , which had not yet been done for the family Squalidae, demonstrated that all 13 PCGs were undergoing purifying selection. A table containing comparison information can be found in Table S3. Maximum‐likelihood and Bayesian inference phylomitogenomic analysis supported the monophyly of both the Squalidae family and Squaliformes order. The phylogeny also supported two distinct clades within the genus *Squalus*. The newly assembled mitochondrial genome of *Squalus cubensis* will assist in gathering information vital to ocean preservation and marine species conservation, including accurate identification of specimens and gaining a better understanding of the genomic data gathered with biomonitoring programs based on environmental DNA (eDNA).

## Author Contributions

**Katie Skufca:** data curation (lead), formal analysis (lead), investigation (lead), project administration (supporting), writing – original draft (lead), writing – review and editing (lead). **J. Antonio Baeza:** conceptualization (supporting), funding acquisition (lead), investigation (supporting), methodology (supporting), project administration (supporting), resources (supporting), supervision (supporting), writing – original draft (supporting), writing – review and editing (supporting).

## Conflicts of Interest

The authors declare no conflicts of interest.

## Supporting information


Data S1.

## Data Availability

Sequence data is available online on NCBI GenBank under the accession number OP056876. .

## References

[ece371412-bib-0001] Almerón‐Souza, F. , C. Sperb , C. L. Castilho , et al. 2018. “Molecular Identification of Shark Meat From Local Markets in Southern Brazil Based on DNA Barcoding: Evidence for Mislabeling and Trade of Endangered Species.” Frontiers in Genetics 9: 138. 10.3389/fgene.2018.00138. 29755504 PMC5934587

[ece371412-bib-0002] Arab, M. A. , C. H. zu Siederdissen , K. Tout , A. H. Sahyoun , P. F. Stadler , and M. Bernt . 2017. “Accurate Annotation of Protein‐Coding Genes in Mitochondrial Genomes.” Molecular Phylogenetics and Evolution 106: 209–216. 10.1016/j.ympev.2016.09.024. 27693569

[ece371412-bib-0003] Baeza, J. A. 2022. “An Introduction to the Special Section on Crustacean Mitochondrial Genomics: Improving the Assembly, Annotation, and Characterization of Mitochondrial Genomes Using User‐Friendly and Open‐Access Bioinformatics Tools, With Decapod Crustaceans as an Example.” Journal of Crustacean Biology 42, no. 1: ruac012. 10.1093/jcbiol/ruac012.

[ece371412-bib-0004] Baeza, J. A. , N. C. Stephens , A. Baker , et al. 2024. “Insights Into the Nuclear and Mitochondrial Genome of the Lemon Shark *Negaprion brevirostris* Using Low‐Coverage Sequencing: Genome Size, Repetitive Elements, Mitochondrial Genome, and Phylogenetic Placement.” Gene 894: 147939. 10.1016/j.gene.2023.147939. 38572145 PMC10990291

[ece371412-bib-0005] Benson, G. 1999. “Tandem Repeats Finder: A Program to Analyze DNA Sequences.” Nucleic Acids Research 27, no. 2: 573–580. 9862982 10.1093/nar/27.2.573 PMC148217

[ece371412-bib-0006] Bernt, M. , A. Donath , F. Jühling , et al. 2013. “MITOS: Improved De Novo Metazoan Mitochondrial Genome Annotation.” Molecular Phylogenetics and Evolution 69, no. 2: 313–319. 22982435 10.1016/j.ympev.2012.08.023

[ece371412-bib-0007] Bhargava, P. , J. L. Marshall , W. Dahut , et al. 2001. “A Phase I and Pharmacokinetic Study of Squalamine, a Novel Antiangiogenic Agent, in Patients With Advanced Cancers^1^ .” Clinical Cancer Research 7, no. 12: 3912–3919. 11751482

[ece371412-bib-0008] Bikandi, J. , R. San Millán , A. Rementeria , and J. Garaizar . 2004. “ *In Silico* Analysis of Complete Bacterial Genomes: PCR, AFLP‐PCR, and Endonuclease Restriction.” Bioinformatics 20: 798–799. 10.1093/bioinformatics/btg491. 14752001

[ece371412-bib-0009] Bonfil, R. 1997. “Status of Shark Resources in the Southern Gulf of Mexico and Caribbean: Implications for Management.” Fisheries Research 29, no. 2: 101–117. 10.1016/S0165-7836(96)00536-X.

[ece371412-bib-0010] Boore, J. L. , J. R. Macey , and M. Medina . 2005. “Sequencing and Comparing Whole Mitochondrial Genomes of Animals.” Methods in Enzymology 395: 311–348. 10.1016/S0076-6879(05)95019-2. 15865975

[ece371412-bib-0011] Braccini, J. M. , and D. Waltrick . 2019. “Species‐Specific At‐Vessel Mortality of Sharks and Rays Captured by Demersal Longlines.” Marine Policy 99: 94–98. 10.1016/j.marpol.2018.10.033.

[ece371412-bib-0012] Brooks, E. J. , A. M. L. Brooks , A. Williams , et al. 2015. “First Description of Deep‐Water Elasmobranch Assemblages in the Exuma Sound, the Bahamas.” Deep Sea Research Part II: Topical Studies in Oceanography 115: 81–91. 10.1016/j.dsr2.2015.01.015.

[ece371412-bib-0013] Cady, T. , K. E. Bemis , and J. A. Baeza . 2021. “The Mitochondrial Genome of the Endangered Spiny Butterfly Ray *Gymnura altavela* (Linnaeus 1758) (Myliobatiformes: Gymnuridae) Provides Insights Into Cryptic Lineages.” Mitochondrial DNA Part A DNA Mapping, Sequencing, and Analysis 32, no. 5–8: 186–194. 10.1080/24701394.2023.2251577. 37668057

[ece371412-bib-0014] Capella‐Gutiérrez, S. , J. M. Silla‐Martínez , and T. Gabaldón . 2009. “trimAl: A Tool for Automated Alignment Trimming in Large‐Scale Phylogenetic Analyses.” Bioinformatics 25, no. 15: 1972–1973. 10.1093/bioinformatics/btp348. 19505945 PMC2712344

[ece371412-bib-0015] Chen, H. , X. Chen , H. Yu , and W. Ai . 2016. “Complete Mitochondrial Genome and the Phylogenetic Position of the Taiwan Spurdog Shark *Squalus formosus* (Squaliformes: Squalidae).” Mitochondrial DNA Part B: Resources 1, no. 1: 419–420. 10.1080/23802359.2016.1176884. 33473504 PMC7800233

[ece371412-bib-0016] Churchill, D. A. , M. R. Heithaus , J. J. Vaudo , R. D. Grubbs , K. Gastrich , and J. I. Castro . 2015. “Trophic Interactions of Common Elasmobranchs in Deep‐Sea Communities of the Gulf of Mexico Revealed Through Stable Isotope and Stomach Content Analysis.” Deep Sea Research Part II: Topical Studies in Oceanography 115: 92–102. 10.1016/j.dsr2.2014.10.011.

[ece371412-bib-0017] Cotton, C. F. , D. Derrick , K. Herman , N. Pacoureau , and N. K. Dulvy . 2020. “*Squalus cubensis*. The IUCN Red List of Threatened Species 2020: e. T61416A3104105.” 10.2305/IUCN.UK.2020-3.RLTS.T61416A3104105.en.

[ece371412-bib-0018] Cruz‐Nuñez, G. , H. Palmadóttir , R. Jónsdóttir , and E. Garcia‐Rodríguez . 2009. “Quality of Cuban Shark Liver Oil. Comparison With Icelandic Cod Liver Oil (Calidad Del Aceite de Hígado de Tiburón Cubano. Comparación Con el Aceite de Hígado de Bacalao Islandés).” Revista Electronica de Veterinaria 10, no. 2: 1–10.

[ece371412-bib-0019] Cucini, C. , C. Leo , N. Iannotti , et al. 2021. “EZmito: A Simple and Fast Tool for Multiple Mitogenome Analyses.” Mitochondrial DNA Part B Resources 6, no. 3: 1101–1109. 10.1080/23802359.2021.1899865. 33796755 PMC7995877

[ece371412-bib-0020] Darriba, D. , G. L. Taboada , R. Doallo , and D. Posada . 2011. “ProtTest‐HPC: Fast Selection of Best‐Fit Models of Protein Evolution.” In Euro‐Par 2010 Parallel Processing Workshops. Euro‐Par 2010. Lecture Notes in Computer Science, edited by M. R. Guarracino , vol. 6586. Springer. 10.1007/978-3-642-21878-1_22.

[ece371412-bib-0021] Donath, A. , F. Jühling , M. Al‐Arab , et al. 2019. “Improved Annotation of Protein‐Coding Genes Boundaries in Metazoan Mitochondrial Genomes.” Nucleic Acids Research 47, no. 20: 10543–10552. 10.1093/nar/gkz833. 31584075 PMC6847864

[ece371412-bib-0022] Emerson, M. V. , and A. K. Lauer . 2008. “Current and Emerging Therapies for the Treatment of Age‐Related Macular Degeneration.” Clinical Ophthalmology 2, no. 2: 377–388. 10.2147/opth.s1485. 19668729 PMC2693977

[ece371412-bib-0023] Espinoza, R. , D. Chapman , J. Morris , et al. 2024. “Characteristics and Species Composition of a Small‐Scale Shark Fishery in Puerto Rico: Jurisdictional Issues Enable Legal Landings of Prohibited and Endangered Species.” Fisheries Research 272: 106936. 10.1016/j.fishres.2023.106936.

[ece371412-bib-0024] Ferrari, A. , S. Di Crescenzo , A. Cariani , et al. 2021. “Puzzling Over Spurdogs: Molecular Taxonomy Assessment of the Squalus Species in the Strait of Sicily.” European Zoological Journal 88, no. 1: 181–190.

[ece371412-bib-0025] Finucci, B. , N. Pacoureau , C. L. Rigby , et al. 2024. “Fishing for Oil and Meat Drives Irreversible Defaunation of Deepwater Sharks and Rays.” Science 383, no. 6687: 1135–1141. 38452078 10.1126/science.ade9121

[ece371412-bib-0026] Giovos, I. , R. N. Aga Spyridopoulou , N. Doumpas , et al. 2021. “Approaching the “Real” State of Elasmobranch Fisheries and Trade: A Case Study From the Mediterranean.” Ocean & Coastal Management 211: 105743. 10.1016/j.ocecoaman.2021.105743.

[ece371412-bib-0027] Grant, J. R. , E. Enns , E. Marinier , et al. 2023. “Proksee: In‐Depth Characterization and Visualization of Bacterial Genomes.” Nucleic Acids Research 51: gkad326. 10.1093/nar/gkad326. PMC10320063 37140037

[ece371412-bib-0028] Gruber, A. R. , R. Lorenz , S. H. Bernhart , R. Neuböck , and I. L. Hofacker . 2008. “The Vienna RNA Websuite.” Nucleic Acids Research 36, no. suppl_2, 1: W70–W74. 10.1093/nar/gkn188. 18424795 PMC2447809

[ece371412-bib-0030] Jin, J. J. , W. B. Yu , J. B. Yang , et al. 2020. “GetOrganelle: A Fast and Versatile Toolkit for Accurate De Novo Assembly of Organelle Genomes.” Genome Biology 21: 1–31. 10.1186/s13059-020-02154-5 PMC7488116 32912315

[ece371412-bib-0031] Jones, L. M. , W. B. Driggers III , E. R. Hoffmayer , K. M. Hannan , and A. N. Mathers . 2013. “Reproductive Biology of the Cuban Dogfish in the Northern Gulf of Mexico.” Marine and Coastal Fisheries 5, no. 1: 152–158. 10.1080/19425120.2013.768572.

[ece371412-bib-0032] Jühling, F. , J. Pütz , M. Bernt , et al. 2012. “Improved Systematic tRNA Gene Annotation Allows New Insights Into the Evolution of Mitochondrial tRNA Structures and Into the Mechanisms of Mitochondrial Genome Rearrangements.” Nucleic Acids Research 40, no. 7: 2833–2845. 10.1093/nar/gkr1131. 22139921 PMC3326299

[ece371412-bib-0033] Kemper, J. M. , and G. J. P. Naylor . 2016. “The Complete Mitochondrial Genome and Phylogenetic Position of the Philippines Spurdog, *Squalus montalbani* .” Mitochondrial DNA Part A DNA Mapping, Sequencing, and Analysis 27, no. 6: 4522–4523. 10.3109/19401736.2015.1101544. 26539656

[ece371412-bib-0034] Kerpedjiev, P. , S. Hammer , and I. L. Hofacker . 2015. “Forna (Force‐Directed RNA): Simple and Effective Online RNA Secondary Structure Diagrams.” Bioinformatics 31, no. 20: 3377–3379. 26099263 10.1093/bioinformatics/btv372 PMC4595900

[ece371412-bib-0035] Kiser, H. , K. Skufca , K. E. Bemis , and J. A. Baeza . 2024. “Comparative Analysis of the Mitochondrial Genomes of Smoothhound Sharks Provide Insight Into the Phylogenetic Relationships Within the Family Triakidae.” Gene Reports 36: 101957. 10.1016/j.genrep.2024.101957.

[ece371412-bib-0036] Kousteni, V. , S. Mazzoleni , K. Vasileiadou , and M. Rovatsos . 2021. “Complete Mitochondrial DNA Genome of Nine Species of Sharks and Rays and Their Phylogenetic Placement Among Modern Elasmobranchs.” Genes 12, no. 324. 10.3390/genes12030324. PMC7995966 33668210

[ece371412-bib-0037] Leary, A. E. 2015. “Effects of the Deepwater Horizon Oil Spill on Deep Sea Fishes.” UNF Graduate Theses and Dissertations. https://digitalcommons.unf.edu/etd/566.

[ece371412-bib-0038] Lee, B. D. 2018. “Python Implementation of Codon Adaptation Index.” Journal of Open Source Software 3, no. 30: 905. 10.21105/joss.00905.

[ece371412-bib-0059] Li, J. , Z. Zhang , S. Vang , et al. 2009. “Correlation Between Ka/Ks and Ks is Related to Substitution Model and Evolutionary Lineage.” Journal of Molecular Evolution 68: 414–423. 19308632 10.1007/s00239-009-9222-9

[ece371412-bib-0039] Limbocker, R. , S. Errico , D. Barbut , et al. 2022. “Squalamine and Trodusquemine: Two Natural Products for Neurodegenerative Diseases, From Physical Chemistry to the Clinic.” Natural Product Reports 39, no. 4: 742–753. 10.1039/d1np00042j. 34698757

[ece371412-bib-0040] Lorenz, R. , S. H. Bernhart , C. Höner zu Siederdissen , et al. 2011. “ViennaRNA Package 2.0.” Algorithms for Molecular Biology 6, no. 1: 26. 22115189 10.1186/1748-7188-6-26 PMC3319429

[ece371412-bib-0041] Nguyen, L. T. , H. A. Schmidt , A. Von Haeseler , and B. Q. Minh . 2015. “IQ‐TREE: A Fast and Effective Stochastic Algorithm for Estimating Maximum‐Likelihood Phylogenies.” Molecular Biology and Evolution 32, no. 1: 268–274. 10.1093/molbev/msu300. 25371430 PMC4271533

[ece371412-bib-0042] Pfleger, M. O. 2016. “Taxonomy and Phylogeography of Deep Sea Dogfish Sharks.” [University of West Florida] https://ircommons.uwf.edu/esploro/outputs/graduate/TAXONOMY‐AND‐PHYLOGEOGRAPHY‐OF‐DEEP‐SEA/99380090733806600.

[ece371412-bib-0043] Ronquist, F. , M. Teslenko , P. Van der Mark , et al. 2012. “MrBayes 3.2: Efficient Bayesian Phylogenetic Inference and Model Choice Across a Large Model Space.” Systematic Biology 61, no. 3: 539–542. 10.1093/sysbio/sys029. 22357727 PMC3329765

[ece371412-bib-0044] Ruiz‐Abierno, A. , J. F. Márquez‐Farías , A. Rojas‐Corzo , V. Miller , J. A. Angulo‐Valdés , and R. E. Hueter . 2021. “Seasonal Abundance and Size Structure of Sharks Taken in the Pelagic Longline Fishery Off Northwestern Cuba.” Marine and Coastal Fisheries 13: 275–291. 10.1002/mcf2.10152.

[ece371412-bib-0045] Shipley, O. N. , L. A. Howey , E. R. Tolentino , L. K. B. Jordan , and E. J. Brooks . 2017. “Novel Techniques and Insights Into the Deployment of Pop‐Up Satellite Archival Tags on a Small‐Bodied Deep‐Water Chondrichthyan.” Deep Sea Research Part I: Oceanographic Research Papers 119: 81–90. 10.1016/j.dsr.2016.11.005.

[ece371412-bib-0046] Shipley, O. N. , N. V. C. Polunin , S. P. Newman , et al. 2017. “Stable Isotopes Reveal Food Web Dynamics of a Data‐Poor Deep‐Sea Island Slope Community.” Food Webs 10: 22–25. 10.1016/j.fooweb.2017.02.004.

[ece371412-bib-0047] Sievers, F. , and D. G. Higgins . 2014. “Clustal Omega, Accurate Alignment of Very Large Numbers of Sequences.” Methods in Molecular Biology 1079: 105–116. 10.1007/978-1-62703-646-7_6. 24170397

[ece371412-bib-0048] Stothard, P. 2000. “The Sequence Manipulation Suite: JavaScript Programs for Analyzing and Formatting Protein and DNA Sequences.” BioTechniques 28: 1102–1104. 10868275 10.2144/00286ir01

[ece371412-bib-0049] Tagliafico, A. , S. Rangel , and M. K. Broadhurst . 2019. “Maturation and Reproduction of Squalus Cubensis and Squalus Cf. Quasimodo (Squalidae, Squaliformes) in the Southern Caribbean Sea.” Ichthyological Research 66: 1–8. 10.1007/s10228-018-0640-9.

[ece371412-bib-0050] Talwar, B. , E. J. Brooks , J. W. Mandelman , and R. D. Grubbs . 2017. “Stress, Post‐Release Mortality, and Recovery of Commonly Discarded Deep‐Sea Sharks Caught on Longlines.” Marine Ecology Progress Series 582: 147–161. 10.3354/meps12334.

[ece371412-bib-0051] Tamura, K. , G. Stecher , and S. Kumar . 2021. “MEGA11: Molecular Evolutionary Genetics Analysis Version 11.” Molecular Biology and Evolution 38: 3022–3027. 33892491 10.1093/molbev/msab120 PMC8233496

[ece371412-bib-0052] Veríssimo, A. , D. Zaera‐Perez , R. Leslie , et al. 2017. “Molecular Diversity and Distribution of Eastern Atlantic and Mediterranean Dogfishes Squalus Highlight Taxonomic Issues in the Genus.” Zoologica Scripta 46, no. 4: 414–428.

[ece371412-bib-0053] Wang, D. , Y. Zhang , Z. Zhang , J. Zhu , and J. Yu . 2010. “KaKs_Calculator 2.0: A Toolkit Incorporating Gamma‐Series Methods and Sliding Window Strategies.” Genomics, Proteomics & Bioinformatics 8, no. 1: 77–80. 10.1016/S1672-0229(10)60008-3. PMC5054116 20451164

[ece371412-bib-0054] Wang, D. P. , H. L. Wan , S. Zhang , and J. Yu . 2009. “γ‐MYN: A New Algorithm for Estimating Ka and Ks With Consideration of Variable Substitution Rates.” Biology Direct 4: 20. 10.1186/1745-6150-4-20. 19531225 PMC2702329

[ece371412-bib-0055] Watanabe, Y. I. , T. Suematsu , and T. Ohtsuki . 2014. “Losing the Stem‐Loop Structure From Metazoan Mitochondrial tRNAs and Co‐Evolution of Interacting Factors.” Frontiers in Genetics 5: 83261. 10.3389/fgene.2014.00109 PMC4013460 24822055

[ece371412-bib-0056] Yang, L. , K. A. Matthes‐Rosana , and G. J. P. Naylor . 2016. “Determination of Complete Mitochondrial Genome Sequence From the Holotype of the Southern Mandarin Dogfish *Cirrhigaleus Australis* (Elasmobranchii: Squalidae).” Mitochondrial DNA Part A DNA Mapping, Sequencing, and Analysis 27, no. 1: 593–594. 10.3109/19401736.2014.908360. 24725011

[ece371412-bib-0057] Zhang, N. , H.‐Y. Guo , L. Guo , et al. 2019. “Characterization of the Complete Mitochondrial Genome of *Squalus brevirostris* (Squaliformes, Squalidae).” Mitochondrial DNA Part B Resources 4, no. 2: 2902–2903. 10.1080/23802359.2019.1660595. 33365783 PMC7706783

[ece371412-bib-0058] Zhu, K. C. , Y. Y. Liang , N. Wu , et al. 2017. “Sequencing and Characterization of the Complete Mitochondrial Genome of Japanese Swellshark (*Cephalloscyllium umbratile*).” Scientific Reports 7: 15299. 10.1038/s41598-017-15702-0. 29127415 PMC5681689

