# Supplementary material for: The Complete Mitochondrial Genome of Squalus cubensis and Comparative Mitogenomics and Phylomitogenomics of the Family Squalidae
Source: Ecol Evol. 2025 May 6;15(5):e71412. doi: 10.1002/ece3.71412 (PMC12055450; doi:10.1002/ece3.71412)
Supplement: Supplementary file 1 — Data S1. [file ECE3-15-e71412-s001.pdf]

Supplementary Material

Table S1: Nucleotide usage of the mitochondrial genomes within the family Squalidae, calculated using MEGA 11.

**Species name**	**A%**	**T%**	**G%**	**C%**	**AT%**
Squalus cubensis	30.90	30.00	14.30	24.80	60.90
Squalus brevirostris	30.70	30.40	14.30	24.60	61.10
Squalus formosus	30.90	30.50	14.20	24.50	61.40
Squalus blainville	30.80	30.09	14.35	24.76	60.89
Squalus montalbani	30.80	30.40	14.30	24.60	61.20
Squalus acanthias	30.80	30.40	14.30	24.50	61.20
Cirrhigaleus australis	30.70	30.50	14.30	24.50	61.20

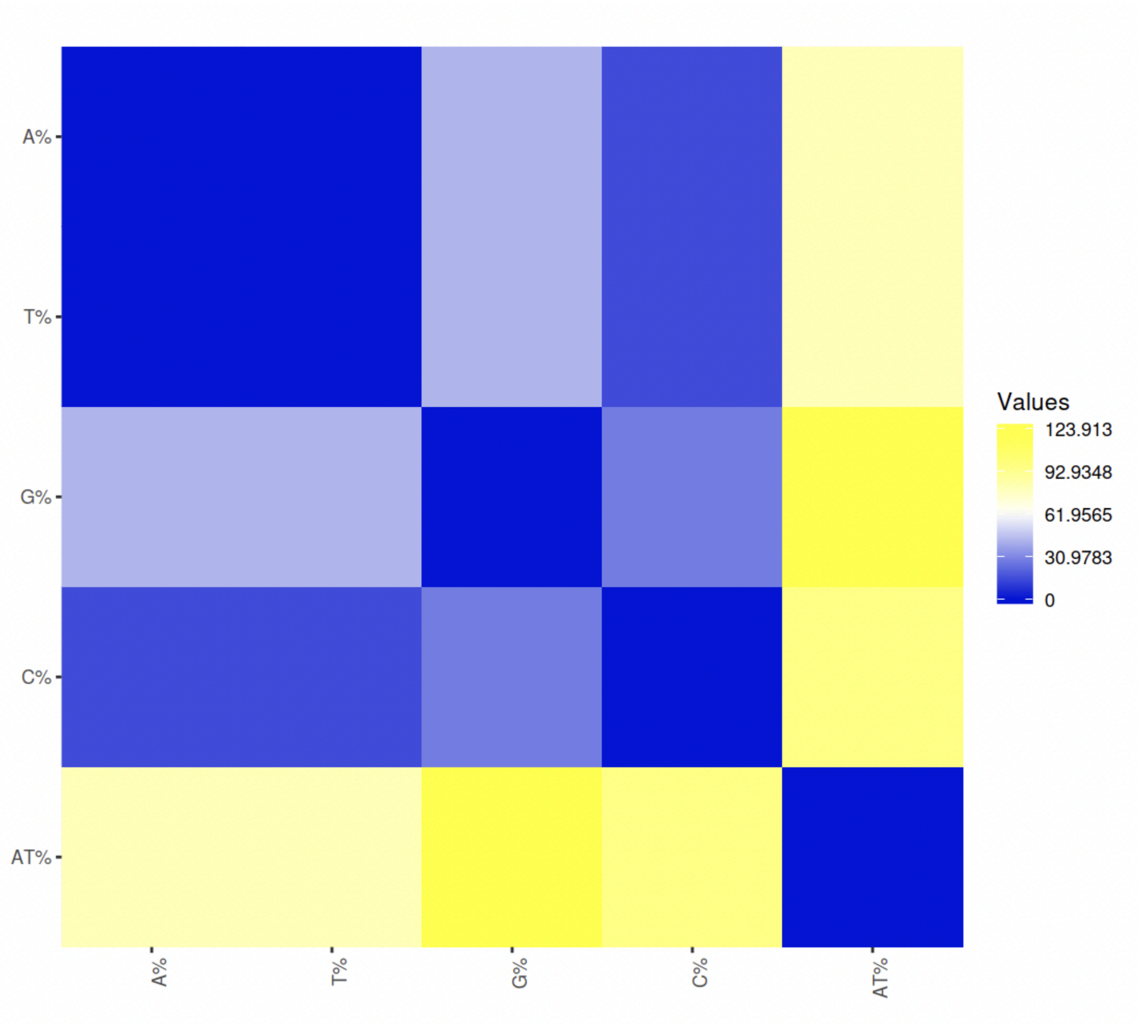

Figure S1: Heatmap comparison of nucleotide usage for the Squalidae family

Table S2: Microsatellites found within the control region of the mitochondrial genome of *Squalus cubensis*, predicted using BioPHP Microsatellite Repeats Finder.

**Position**	**Cycle**	**Repeats**	**Sequence**
20	2	4	ATATATAT
64	2	3	ATATAT
81	2	3	ATATAT
210	2	3	TTTTTT
508	2	5	TTTTTTTTTT
861	2	3	GGGGGG
896	2	5	TTTTTTTTTT
915	2	3	CCCCCC
923	2	3	CCCCCC
984	2	5	TTTTTTTTTT

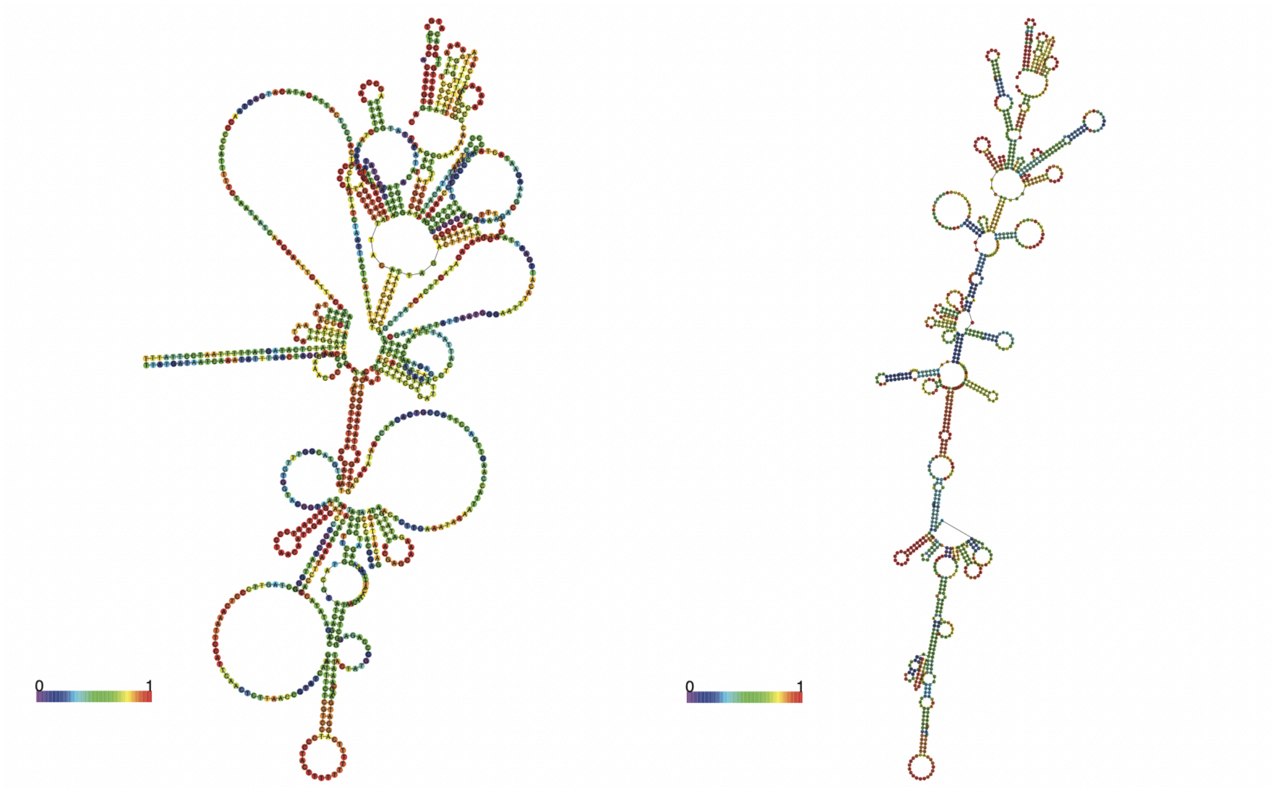

Figure S2: Predictions of the structure of the control region within the mitochondrial genome of *Squalus cubensis* (Centroid, left; Minimum free energy, right).

Table S3: Comparison of mitogenome features across the family Squalidae.

Species name	Squalus cubensis	Squalus brevirostris	Squalus formosus	Squalus blainville	Squalus montalbani	Cirrhigaleus australis	Squalus acanthias
Genbank Number	OP056876	KY111436	KU951280	MT274575	KT459334	KJ128289	NC_002012
Mitogenome Lenght	16,753	16,734	16,735	16,705	16,555	16,544	16,738
A%	30.90%	30.70%	30.90%	30.80%	30.80%	30.70%	30.80%
T%	30.00%	30.40%	30.50%	30.09%	30.40%	30.50%	30.40%
G%	14.30%	14.30%	14.20%	14.35%	14.30%	14.30%	14.30%
C%	24.80%	24.60%	24.50%	24.76%	24.60%	24.50%	24.50%
AT%	60.90%	61.10%	61.40%	60.89%	61.20%	61.20%	61.20%
Truncated Serine	S1	S1	S1	S1	S1	S1	n/a
Cloverleaf Serine	S2	S2	S2	S2	S2	S2	n/a
Longest PCG	Atp8 (168 bp)	168 bp	n/a	Atp8 (168 bp)	n/a	Atp8 (168 bp)	n/a
Shortest PCG	Nad5 (1833 bp)	1833 bp	n/a	Nad5 (1833 bp)	n/a	Nad5 (1833 bp)	n/a
Most used PCG codons	ATT, TTA, CTA	n/a	n/a	ATT, TTA, TTT	n/a	n/a	n/a
Least used PCG codons	TAG, GCG, CGG	n/a	n/a	GCG, CGG, CGT	n/a	n/a	n/a
Highest RSCU PCG	CGA (2.222)	n/a	n/a	UUA (1.57)	n/a	n/a	n/a
Lowest RSCU PCG	GCG (0.056)	n/a	n/a	GCG (0.13)	n/a	n/a	n/a
Highest Ka/Ks PCG	0.0762 (Nad4l)	n/a	n/a	n/a	n/a	n/a	n/a
Lowest Ka/Ks PCG	3.41 x 10^-15 (Nad3)	n/a	n/a	n/a	n/a	n/a	n/a
Longest tRNA	Leucine 2 (75 bp)	n/a	n/a	Leucine 2 (75 bp)	n/a	Leucine 2 (75 bp)	n/a
Shortest tRNA	Cysteine, Serine 1 (67 bp)	n/a	n/a	Cysteine, Serine 1 (67 bp)	n/a	Cysteine, Serine 1 (67 bp)	n/a
Bp lenght of small (12S) rRNA	953	n/a	951	951	n/a	954	n/a
Bp lenght of large (16S) rRNA	1653	n/a	1676	1677	n/a	1678	n/a
Bp lenght of CR	1049	n/a	1079	1096	n/a	885	n/a
CR located between	tRNA Phe, tRNA Pro	tRNA Phe, tRNA Pro	tRNA Phe, tRNA Pro	tRNA Phe, tRNA Pro	n/a	tRNA Phe, tRNA Pro	n/a
AT% Skew of CR	66.80%	n/a	66.90%	n/a	n/a	n/a	n/a
Tandem repeats in CR	None	n/a	n/a	None	n/a	n/a	n/a
# of Microsatellite repeats in CR	10	n/a	n/a	n/a	n/a	n/a	n/a
